# Identification of Barriers and Needs in the Discontinuation of Benzodiazepine Receptor Agonists in Elderly Patients of a Rural Community—A Qualitative Study

**DOI:** 10.3390/geriatrics10010018

**Published:** 2025-02-01

**Authors:** Tiago de Barros Mendes, Marta Nazha, Ana Luísa Neves, Paula Broeiro-Gonçalves

**Affiliations:** 1 USF Portel (ULS Alentejo Central), Parque da Matriz, 7220-361 Portel, Portugal; 2 School of Health and Human Development (SHHD), University of Évora, Colégio Luis António Verney, Rua Romão Ramalho, nº59, 7000-671 Evora, Portugal; 3 USF São Filipe (ULS Arrábida), Rua Batalha do Viso, nº 46, 2900-264 Setubal, Portugal; marta.nazha@gmail.com; 4 Department of Primary Care and Public Health, Imperial College London, London W6 8RP, UK; ana.luisa.neves14@imperial.ac.uk; 5 Faculty of Medicine, The University of Porto, Rua Dr. Plácido da Costa, s/n, 4200-450 Oporto, Portugal; 6 NOVA Medical School, Universidade Nova de Lisboa, Campo Mártires da Pátria, nº 130, 1169-056 Lisbon, Portugal; paulabroeiro@gmail.com; 7 UCSP Olivais (ULS Lisboa Central), Alameda da Encarnação, Olivais, 1800-192 Lisbon, Portugal

**Keywords:** benzodiazepines, deprescription, geriatrics, qualitative research

## Abstract

**Background/Objectives**: The predictors of successful discontinuation of benzodiazepine agonist receptors (BZRA) in elderly patients are not well known due to lack of research on the subject, and there is a need for further investigation, with more focus from the patients’ point of view. No previous studies were identified that have been conducted in Portugal on this subject. We proposed to identify the barriers and facilitators in the discontinuation of BZRA from the perceptions of elderly patients under prolonged prescription of BZRA, belonging to the same rural community. The contributions for further research are intended to be the identification of potential intervention targets directed at patients to reduce the prevalence of elderly patients under prolonged prescription of BZRA. **Methods**: A set of 15 semi-structured interviews with patients under prolonged prescription of BZRA was conducted. Content analysis was done by the main researcher and a reviewer to identify original emerging themes for the two underlying domains. **Results**: Four themes were identified as barriers to the discontinuation of BZRA: (1) patient characteristics, (2) clinical factors, (3) medication-related factors, and (4) context and external factors. Seven themes were identified as facilitators to the discontinuation of BZRA: (1) motivation, (2) patients’ knowledge, (3) perception of BZRA insufficiency, (4) access to written information, (5) access to alternatives, (6) time for decision-making, and (7) attitudes of health professionals. **Conclusions**: The findings highlight the challenging nature of BZRA discontinuation and the range of barriers and facilitators that impact patients’ behaviour towards this purpose. We subdivided the elements identified in two areas, therefore aiming at producing significant knowledge to outline potential intervention targets.

## 1. Introduction

As part of the monitoring of the Portuguese mental health program, the indicator “proportion of users aged 65 and over, without prolonged prescription of anxiolytics, sedatives, or hypnotics” was defined [1], which reveals the importance provided to this topic in Portugal.

The drugs in question are benzodiazepines and analogues (e.g., zolpidem), in the scope of this study referred to as Benzodiazepine Receptor Agonists (BZRA). According to the description of the indicator referred to, prolonged prescription is understood as the sum of an Average Daily Maintenance Dose > 53 in the last 12 months. This value is estimated according to the maximum thresholds foreseen for the treatment of pathological anxiety or insomnia, adjusted for the elderly population, according to the Clinical Practice Guideline nº 55/2011 of the Portuguese Directorate-General of Health (DGS) [2].

BZRA is the most prescribed group of drugs used to treat symptomatic anxiety, as well as primary and secondary sleep disorders. Although well-tolerated, their use has been restricted due to the risk of dependence and habituation in a considerable number of users, leading to marked difficulty in discontinuing them in users who have been taking them for a few weeks [2].

Alongside the high prevalence of anxiety syndromes, Portugal has one of the highest prescription rates of BZRA in Europe. Recent reports show that these drugs are responsible for 7% of the drug market share in the country [3].

Despite the existing evidence on the increased risk of these therapies in this age group, the major consumers of BZRA tend to be older patients and, in general, their consumption increases with ageing. Recent estimates of prevalence in the older population throughout the developed world have shown a rate of approximately 8–12% in community-based studies [4].

Although their use may be indicated in certain circumstances, most prescriptions of BZRA for the elderly are considered inappropriate. Pharmacological treatment with BZRA in elderly patients is associated with an increased risk of falls, balance problems, drowsiness, cognitive impairment, memory disorders, functional impairment, and physical dependence [5].

Therefore, understanding patient perspectives more in-depth may help identify and provide a foundation for the development of interventions with greater reach, sustainability, and effectiveness in primary care [5,6]. In this sense, it is crucial to develop tools that help the discontinuation of BZRA in the elderly to prevent its possible negative consequences. However, the predictors of successful discontinuation are not well known due to a lack of research on the subject [4,5,6,7].

Deprescription is defined as ‘‘the withdrawal of an inappropriate medication, supervised by a health care professional with the goal of managing polypharmacy and improving outcomes’’ [4].

While studies have identified several barriers, including dependence, withdrawal symptoms, and lack of supports, there are gaps in comprehensive analysis of both barriers and facilitators, as well as in comparing the experiences of current and previous long-term BZRA users. To ensure that future interventions target behavioral determinants and provide theoretical underpinning, discontinuation of long-term BZRA use needs to be studied with more focus from the patients’ point of view [8].

In developing new interventions aimed at BZRA deprescribing, it is increasingly recommended that researchers adopt a systematic approach and explicitly describe the process of its development [8].

A systematic review by Rasmussen et al. in 2021 [5] aimed to identify and compare barriers and facilitators in stakeholders involved in BZRA deprescribing in the elderly (patients, physicians, nurses, and caregivers) to identify potential gaps in this field of research.

Eight themes emerged from this review defined as barriers to BZRA deprescribing in patient-directed studies: understanding BZRA as an effective treatment; understanding BZRA as an inducer of well-being; thinking that BZRA is not harmful; concern about withdrawal symptoms; ageism (understanding unnecessary de-escalation due to age); lack of information (on side effects, dependency, and alternative treatments); dependence (feeling unable to reduce or stop therapy); and lack of medical support.

Five themes emerged that were defined as facilitators for BZRA deprescribing in patient-directed studies: educational tools (e.g., brochures); motivation of health professionals; perception of side effects; willingness to stop therapy; and time to consider the benefits after counselling in the face of deprescribing.

The same review points in its conclusions to the importance of communication and shared decision-making between doctors and patients to improve medication according to patient preferences, as well as the need for further research on this topic.

A systematic review and meta-synthesis of patients’ experiences and perceptions of seeking and using BZRA, published in 2016 [9], points out seven themes from published qualitative research, which relate to factors perpetuating their use: patients’ negative perceptions of insomnia and its impact, failed self-care strategies, triggers to medical help-seeking, attitudes towards treatment options/service provision, varying patterns of use, withdrawal, and reasons for initial or ongoing use. The authors point out three main strategies that may support patients with conditions for which these drugs may be prescribed: creating educational resources (implementation of self-care strategies, helping patients to have realistic expectations of what treatments might achieve), providing alternative treatments (such as cognitive behavioral therapy), and expanding the range of help options to explore and encourage the use of non-pharmacological treatments, ensuring that patients know that their problems are taken seriously. Further research is needed to ascertain the efficacy of different approaches to deprescribing (e.g., patient education, group therapy, and mixed interventions). Moreover, it is important to consider the influence resulting from patient characteristics (e.g., comorbidities).

Intervention studies on deprescribing show that BZRA withdrawal seems to be feasible and safe in the elderly population despite heterogeneity in the study methodology and outcomes [4], which has been substantiated by three additional systematic reviews on the evidence for interventions to reduce or discontinue BZRA in this age group [10,11,12].

A review article by Ng et al. [4], related to the impact of interventions aimed at BZRA deprescribing in elderly patients, reports quite different success rates (ranging from 27 to 80%). This variability is assignable to the heterogeneity of intervention methodologies, as well as limited applicability to patients with cognitive impairment. They distinguished two main intervention typologies targeting patients, each of them grouping different interventional strategies: Raising Awareness Interventions consisted of: written information (patient information book), minimal intervention or geriatrician one-off counselling; Providing Resources for BZRA Discontinuation consisted of: gradual self-managed dose reduction, supervised gradual dose reduction, and supervised gradual dose reduction plus cognitive behavioural therapy, as well as other techniques.

Oliveira et al. [3] summarize, among other topics concerning BZRA withdrawal in primary health care, available strategies to discontinue these drugs at this setting. It points out that the success of discontinuing therapy in long-term users seems to be associated with the perception of self-efficacy, beliefs about withdrawal symptoms, initial motivation to quit, information about the risks of BZRA, and gradual dose-decreasing withdrawal.

Related qualitative studies have examined patients’ experiences and perceptions of BZRA use. Very few peer-review studies have considered benzodiazepines from the user perspective, and even fewer have drawn on the language employed by users themselves to explore firsthand experiences of withdrawal or discontinuation syndrome [13].

A study conducted in Ireland by Lynch et al. [8], consisting of semi-structured interviews based on the Theoretical Domains Framework with people with current or previous experience of prolonged BZRA use, highlights the complexity of BZRA discontinuation, identifying a wide range of mediators (barriers and facilitators) of discontinuing long-term BZRA use from the perspective of both current and previous users. They propose that future work will involve developing a theory-based intervention that may support BZRA discontinuation in primary health care.

A study conducted in Canada by Allary et al. [14], designed to figure out psychological predictors of benzodiazepine among older adults, points out three psychological factors that were significant predictors for BZRA discontinuation: social support satisfaction, intensity of depressive symptoms, and self-perceived competence in the ability to withdraw. According to the authors, the importance of social support satisfaction and self-perceived competence should result in future BZRA withdrawal programs focusing more on these factors to be more effective, as it appears to be the best way to achieve long-term discontinuation.

A recent qualitative study in primary health care centres in an interior and mostly rural region of Portugal (similar to the context of our study) explored general practitioners’ perspectives on solutions to address the excessive prescription of benzodiazepines [15]. Solutions proposed focused on organizational aspects, alternative approaches to prescribing, and wider community-based initiatives (such as fostering public awareness about the perils of BZRA, creating socially adapted consultations, and more community-based programs that would enable the pursuit of therapeutic non-pharmacologic alternatives).

In this sense, and having not found in the literature review any similar qualitative studies conducted in Portugal to date, we proposed to identify the barriers and facilitators in the discontinuation of BZRA from the perceptions of elderly patients under the prolonged prescription of BZRA, belonging to the same rural community.

The potential contributions for further research are the identification of intervention targets directed at patients to reduce the prevalence of elderly patients under prolonged prescription of BZRA.

## 2. Materials and Methods

### 2.1. Design

Since the main purpose of the study was to identify individual perceptions on this topic, we considered that the qualitative methodology would be the most proper to conduct this investigation. Therefore, the aim was to conduct a set of semi-structured interviews headed from an interview guide drawn up based on literature to then conduct the respective content analysis.

### 2.2. Preparation of the Interview Guide

An interview topic guide with open-ended questions was developed based on eight domains called “Barriers to the Deprescribing of BZRA” and five domains called “Facilitators to the Deprescribing of BZRA”, according to the themes identified in the systematic review by Rasmussen et al. [5].

The interview guide was reformulated after conducting a pilot test for its experimental application, which consisted of obtaining a written opinion on it, voluntarily requested from two family doctors and two patients who did not comply with the performance indicator of interest to the study (i.e., potentially eligible for inclusion).

### 2.3. Recruitment

A convenience non-probability sampling was obtained (with a minimum quota of 25% by gender) from the population of patients registered in the Family Health Unit Matriz (Arraiolos Health Centre, Alentejo Region, Portugal). The inclusion criteria were: not accomplish the indicator “proportion of users aged 65 years or over, without prolonged prescription of anxiolytics, sedatives or hypnotics”, according to the respective list obtained, relative to December 2021, from the database made available by the software MIM@UF—Module for Information and Monitoring of Functional Units (version 2021). As exclusion criteria, we defined: (a) registration in the Electronic Health Record’s Active Problems List of codes N88 (Epilepsy) and/or P70 (Dementia), according to the ICPC-2 (International Classification of Primary Health Care); (b) severe psychiatric illness (defined as any clinical condition requiring the inclusion of antipsychotic drugs and/or lithium in the patient’s chronic medication list); (c) record of any previous medical consultation conducted by the main researcher (due to the circumstance of being himself a family doctor at the unit where the study was developed), which was intended to cancel a possible effect of “moderator bias” that the existence of a previous doctor–patient relationship may induce; and (d) any circumstances or conditions that would make the interview infeasible to be performed without the presence of a third person.

Prior telephone contact with patients selected, for invitation and scheduling of the interview, was conducted by a participants’ family health unit nurse, who was, therefore, assigned the role of referrer/interlocutor between the main researcher and each study participant.

### 2.4. Data Collection

Semi-structured interviews were conducted by the main researcher with a group of 15 patients from the sampling obtained, with a dimension needed to reach data saturation.

The interviews were conducted at Arraiolos Health Centre in an office usually used for institutional meetings where medical or nursing consultations are not conducted.

Prior to the completion of each interview, the respective informed consent was obtained in duplicate, signed by the participant, interviewer, the scientific supervisors, and the doctor responsible for the unit where the study was conducted, with both interviewer and participant keeping the respective copies. The document pointed out the commitment from the main researcher to the use of the data collected exclusively for the purposes of the study.

In the preliminary phase of each interview, the following general data were recorded for description of participants: (a) gender; (b) age; (c) active principle and dosage of the current BZRA; (d) year of current BZRA’s first prescription; (e) previous intake of other BZRAs; (f) cause for taking BZRA (according to the interviewee); (g) frequency of intake; and (h) previous attempts to discontinue the BZRA carried by the interviewee.

The data collection of the interview was conducted through audio recording by two electronic devices, with voice recording software in mp3 and wav formats.

The transcripts from the recordings made were obtained in full by the main researcher, following the interviews. None were returned to the participants for comments and/or corrections.

A numeric code was assigned to each participant to ensure their anonymity in the study from the stage of obtaining the transcript of the audio recording.

### 2.5. Data Analysis

The main and the collaborating researchers independently went ahead to review the transcripts, using thematic analysis method to identify original emerging themes for the two underlying domains (barriers to discontinuation/facilitators to discontinuation). Thematic analysis data were organized using Microsoft Excel^®^ software (version 2411 Build 16.0).

A second version of the thematic analysis was obtained, reconciling the data obtained by the two researchers by consensus.

A third (definitive) version of the thematic analysis was obtained, adapting the original themes obtained to those previously found in the systematic review by Rasmussen et al. [5].

At every stage of the data analysis process, we intended to understand key themes in the data and how they relate to one another, allowing the ongoing inclusion of emergent themes, which were supported by participants’ quotations. We followed the different phases of thematic analysis [16], generating an initial coding framework that formed the source from which the continuous inclusion of emerging themes and subthemes was conducted, supported by participants’ quotations during the whole analytic process.

The Consolidated Criteria for Reporting Qualitative Studies (COREQ) [17] was used to ensure the study met the recommended standards of qualitative data reporting.

### 2.6. Ethical Considerations

The study was conducted in accordance with the Declaration of Helsinki and approved by the Alentejo’s Regional Health Administration Ethics Committee (Process number, 09/CE/2022; approval date, 12 May 2022; Feedback number, 05/CE/2022). In this regard, and at the request of the ethics committee, the researcher added a safeguard to the study protocol in the event that, during the interview, the perception of some clinical condition or other in the interviewee that could imply, due to professional responsibility, the need for some type of medical intervention, the appropriate referral would be made to the respective family health team, provided that the express consent of the interviewee was obtained, with the exception of legally foreseen circumstances. This eventuality did not occur in any of the 15 interviews conducted. Also, aiming to minimize any possible negative impact on the participant’s health that could result from conducting the interview, the protocol also expressed the possibility that it could be interrupted in the event of any circumstance that could constitute an overload (physical or psychological) to its continuation. This situation also did not occur in any of the interviews conducted.

## 3. Results

### 3.1. Participant Characteristics

Fifteen individual interviews were conducted with selectable patients, the longest lasting 27 min and the shortest 10 min. Each interview was conducted in a single moment, and none of the interviewees withdrew from participating in the study during or after the interview. Eleven of fifteen of those interviewed were women. The median age was 70 years (mean 71.3), ranging from 65 to 82 years of age. All respondents were medicated with only one BZRA, the most frequent being brotizolam 0.25 mg and ethyl loflazepate 2 mg. The time elapsed since the current BZRA prescription ranged from 1 to 22 years, with a median of 8 years (mean 7.6 years). Nine of the interviewees had previously been medicated with other BZRAs other than the current one. The reasons for taking BZRA, according to the interviewee, varied between insomnia and anxiety with equal frequency. Ten of the interviewees were taking BZRA continuously, and six had made previous attempts to discontinue it (Table 1).

### 3.2. Barriers and Facilitators Do Discontinuation of BZRA

After adjusting the data obtained individually by each of the two investigators in the content analysis performed, about the barriers to discontinuation of BZRA, four themes emerged, subdivided into eleven specific sub-themes. Themes identified were:

1. Patient characteristics, subdivided into subtheme (1.a.) prejudice of age.
2. Clinical factors, subdivided into subthemes (2.a.) chronicity of the psychological condition related to the use of BZRA; (2.b.) bad sleep habits; and (2.c.) perception of brain/nervous system vulnerability.
3. Medication-related factors, subdivided into subthemes (3.a.) habituation and concern with withdrawal symptoms; (3.b.) valuing the therapeutic benefit of BZRA; and (3.c.) disbelief in a non-pharmacological alternative.
4. Context and external factors, subdivided into subthemes (4.a.) family and social context; (4.b.) community context/prejudices towards mental health; (4.c.) lack of medical support; and (4.d.) other anxiety inducers.

Regarding the facilitators for discontinuation of BZRA, seven themes and six sub-themes emerged. Themes identified were:

1. Motivation to stop BZRA.
2. Patient’s knowledge about BZRA, subdivided into subthemes (2.a.) negative/side effects of BZRA; and (2.b.) benefits of discontinuing medication.
3. Perception of BZRA insufficiency regarding the therapeutic goal.
4. Access to written information.
5. Access to alternatives, subdivided into subthemes (5.a.) pharmacological alternatives; and (5.b.) non-pharmacological alternatives.
6. Time for decision-making.
7. Attitudes of health professionals, subdivided into subthemes (7.a.) competencies; and (7.b.) availability.

Table A1, Table A2, Table A3, Table A4 and Table A5 (Appendix A) compile the identified factors, documented with illustrative quotes selected as the best descriptive for each theme/sub-theme. Each quote is followed by the code assigned to each interviewee.

## 4. Discussion

### 4.1. Summary of Key Findings

This study enabled the identification of barriers and facilitators towards the discontinuation of BZRA from the perspective of elderly patients in a rural community in Portugal, chronically medicated with this pharmacological group.

Four themes emerged as barriers to BZRA deprescribing (patient characteristics; clinical factors; medication-related factors; and context and external factors), subdivided into 11 specific sub-themes.

Seven themes emerged as facilitators to BZRA deprescribing (motivation to stop BZRA; patients’ knowledge about BZRA; perception of BZRA insufficiency regarding the therapeutic goal; access to written information; access to alternatives; time for decision-making; and attitudes of health professionals), subdivided into six specific sub-themes.

### 4.2. Comparison with Previous Literature

Regarding the Barriers to discontinuation of BZRA, four of the identified sub-themes corresponded to themes identified in the systematic review by Rasmussen et al. [5]: 1.a.: prejudice of age; 3.a.: habituation and concern with withdrawal symptoms; 3.b.: valuing the therapeutic benefit of BZRA; and 4.c.: lack of medical support. Seven new sub-themes emerged that did not correspond to those identified in the aforementioned review: 2.a.: chronicity of the psychological condition related to the use of BZRA; 2.b.: bad sleep habits; 2.c.: perception of brain/nervous system vulnerability; 3.c.: disbelief in a non-pharmacological alternative; 4.a.: family and social context; 4.b.: community context/prejudices towards mental health; and 4.d.: other anxiety inducers.

Concerning 1. patient characteristics, other studies point out several topics that did not emerge in this study such as “the existence of an underlying condition forming part of individuals’ identity and necessitating BZRA use” [8], “patients’ negative perceptions of insomnia and its impact and failed self-care strategies” [9].

Regarding 2. clinical factors, chronicity of the psychological condition related to the use of BZRA, (2.a.) is also pointed out by Lynch et al. [8] as “a perceived continued need for BZRA use”. Allary et al. [14] also found “intensity of depressive symptoms” as a barrier to discontinuation.

Respecting 3. medication-related factors, habituation/concern with withdrawal symptoms (3.a.) is also pointed out by themes such as “identifying as being addicted to or dependent on BZRAs”, “negative impact of withdrawal symptoms and lack of continued availability of BZRAs if needed” [8]. Sirdifield et al. [9] underline “the perception from patients of being unable to reduce medication use” and “fear of what would happen if they did discontinue (either based on imagined negative consequences or past experiences)” as additional barriers. Valuing the therapeutic benefit of BZRA (3.b.) is illustrated by Cook et al. [6] as “the attribution to these medications of soothing properties that extend beyond their ordinary use, i.e., affording control over daily stress, bringing tranquility and even prolonging life”. It is also described that “self-reported benzodiazepine dependence was associated with perceptions of (…) feeling reliant on benzodiazepines to be comfortable and able to handle life” [8]. Disbelief in a non-pharmacological alternative (3.c.) is also pointed out by themes such as “availability and commitment required with non-BZRA alternatives” and “effectiveness of alternatives to BZRAs” [8].

About 4. context and external factors, family, and social context (4.a.) is also pointed out by themes such as “encouragement from family members to continue BZRA use” [8] and “importance of social support satisfaction” [14]. Community context/prejudices towards mental health (4.b.) is also illustrated as the “negative information regarding the BZRA discontinuation process posted on online fora" [8]. Lack of medical support (4.c.) is also pointed out by themes such as “access to resources and support for discontinuing BZRA use”, “variation in experience regarding BZRA use/discontinuation with different healthcare professionals”, and “perceived lack of insight into BZRA use and discontinuation methods among GPs” [8]. The “lack of support from GPs”, “a need for specialist withdrawal services”, and the “absence of an appropriate support network” are also described as barriers to discontinuation of BZRA [9].

Relating to the facilitators to the discontinuation of BZRA, five of the identified themes corresponded to those identified in the systematic review by Rasmussen et al. [5]: 1: motivation to stop BZRA; 2: patients’ knowledge about BZRA (subdivided into sub-themes 2.a: negative/side effects of BZRA and 2.b: benefits of discontinuing medication); 4: access to written information; 6: time for decision-making; 7: attitudes of health professionals (subdivided into sub-themes 7.a: competencies and 7.b: availability). Two new themes emerged that did not correspond to those found in the aforementioned review: 3: perception of BZRA insufficiency regarding the therapeutic goal; 5: access to alternatives (subdivided into sub-themes 5.a: pharmacological alternatives and 5.b: non-pharmacological alternatives).

Regarding 1. motivation to stop BZRA, it is also pointed out by themes such as “confidence in the ability to discontinue BZRA use”, “optimistic outlook towards BZRA discontinuation”, “support from family and friends regarding discontinuation of BZRA use” [8], and “self-perceived competence in the ability to withdraw” [14].

With reference to 2. patients’ knowledge about BZRA, the “knowledge of potential risks associated with long-term BZRA use” [8] is also highlighted. Relative to the subtheme negative/side effects of BZRA (2.a.), it is also pointed out that “patients’ knowledge of, and attitudes towards, potential side effects from medication could influence their patterns of use” [8]. Relative to the subtheme benefits of discontinuing medication (2.b.), it is also pointed out as “positive consequences of discontinuing BZRA use” and “the goal of no longer being dependent on BZRA” [8]. It is also highlighted that, among the reasons for withdrawal, is “a realization that the use of these drugs was interfering with their life and the people around them” [9].

No related themes were found in the literature concerning 3. perception of BZRA insufficiency regarding the therapeutic goal and 4. access to written information.

About 5. access to alternatives, “comparable symptomatic relief from any alternative intervention or non-BZRA treatment” and “use of resources and supports to augment dosage reduction process” [8] are also pointed out.

Relating to 6. time for decision-making, the “use of gradual dosage reduction or limiting BZRA intake” [8] is also marked.

Finally, respecting 7. attitudes of health professionals, “the patients’ perception of the attitude of physicians towards continuous benzodiazepine use” is also highlighted as a key factor affecting discontinuation [6]. “Advice and support from healthcare professionals regarding discontinuation of BZRA use” [8] is also supported. Sirdifield et al. [9] mark “attitudes towards treatment options/service provision”, adding, in this regard, that “patients discussed a desire for continuity of care, more discussion with a health professional prior to receiving a prescription, and longer consultation times. Patients wanted more detailed information, and for their problem to be taken seriously”.

### 4.3. Strengths and Limitations of the Study

The relevance of this study stems from previous investigations related to this topic, which underline the need for further investigations, with more focus on the patient’s point of view [4,5,6].

The emerging themes were framed in the information obtained in the literature, upon which the interview guide was built.

All interviews were coded independently by two researchers, which enhanced the robustness of the analysis.

Other possible stakeholders that could have been enrolled in this context (health professionals, caregivers) were not involved in the study.

The results of this study should only be interpreted within the context in which it was developed: a group of patients aged 65 years and over, chronically medicated with BZRA, belonging to the same community in the Alentejo Region (Portugal).

By restricting the sample to patients on the current use of BZRA, we lack the perspective of patients who have successfully previously discontinued this medication.

The fact that only one researcher did the interviews, as well as the lack of previous experience in conducting semi-structured interviews, is a potential bias of the study that may, therefore, have limited the possibility of exploring additional aspects that could be brought up with more experienced interviewers.

### 4.4. Implications for Research

**Figure 1:**
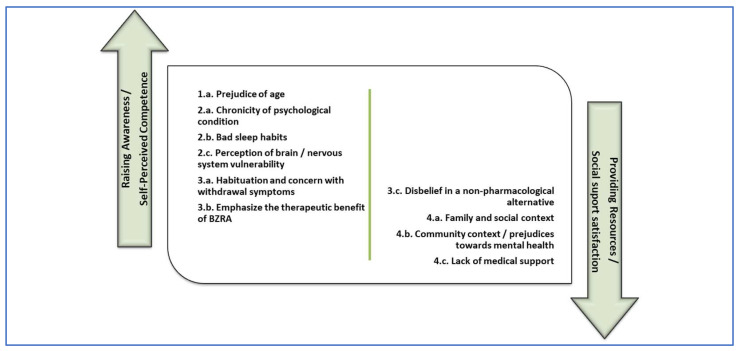
Barriers to discontinuation of BZRA subdivided in potential areas of intervention.

**Figure 2:**
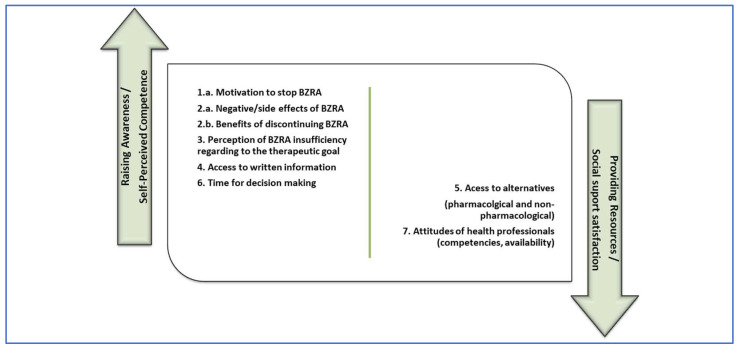
Facilitators to discontinuation of BZRA subdivided in potential areas of intervention.

Figure 1 and Figure 2 are proposals for the distribution of the elements identified as barriers and facilitators from the study, subdivided into two potential areas of intervention. We associated the aforementioned two main intervention typologies indicated by Ng et al. [4], with the two main psychological predictors for BZRA discontinuation pointed out by Allary et al. [14] as main gains for withdrawal programs. Therefore, in the first (left) group, we allocated factors related to raising awareness interventions [4], which we associated with self-perceived competence in the ability to withdraw [14]. In the second (right) group, we allocated factors related to providing resources for BZRA discontinuation [4], which we associated with social support satisfaction [14].

This model may supply an opportunity for the design of a future implementation study in which different intervention components could be involved, such as dissemination among healthcare professionals of validated tools for BZRA discontinuation and health promotion strategies towards the integration of this problem in the community. Examples of these strategies could be intersectional intervention, community participation, and promoting literacy, training, and autonomy of people.

We intend that this study may provide a starting point either for other studies in this area of interest, as for the identification of potential targets for withdrawal programs or interventions, aiming at a more comprehensive approach that can meet the different variables involved in the maintenance of these drugs.

### 4.5. Learnings and Insights

Considering the findings of this study, we may ponder about how suitable it may be to monitor the proportion of elderly patients without long-term prescription of BZRA as a measure of quality of life provision, considering the implications inherent to the socio-family and community context, difficulties in accessing non-pharmacological alternatives and risks inherent to discontinuation in patients with many years of prolonged BZRA intake.

In this sense, it may be important to ponder alternative ways of checking the proportion of elderly patients who were prescribed long-term BZRA. Some alternative possibilities could be to measure new long-term prescription users over a period, rather than in full, and also to value the implementation of local intervention programs towards the discontinuation of BZRA.

## 5. Conclusions

In this study, we intended to explore the experiences and perceptions of elderly patients from a rural community in the Alentejo region (Portugal) under prolonged prescription of BZRA regarding their discontinuation to identify barriers and facilitators that may lead to their chronic usage.

The findings highlight the challenging nature of BZRA discontinuation and the range of barriers and facilitators that may impact patients’ behaviour towards this purpose.

We subdivided the elements identified in two areas of intervention, according to the initial motivation indicated in the Introduction, therefore aiming to produce significant knowledge to outline potential intervention targets to support patients in discontinuing BZRA use to reduce the prevalence of elderly patients under a prolonged prescription of BZRA.

## Figures and Tables

**Table 1:** Characterization data of study participants.

**GENDER**	**[*n* (%)]**
MALE	4 (26.7%)
FEMALE	11 (73.3%)
AGE (years)	[*n* (%)]
65–69	6 (40.0%)
70–74	6 (40.0%)
75–79	0 (0.0%)
≥80	3 (20.0%)
**Descriptive Statistics:** Range [65.82 years]; Median 70 years; Mean 71.3 years	
BZRA IN USE	Active Principle, Dosage
3 participants	Brotizolam 0.25 mg, Ethyl loflazepate 2 mg
2 participants	Diazepam 5 mg
1 participant	Alprazolam 0.5 mg, Bromazepam 3 mg, Clorazepate Dipotassium 5 mg, Cloxazolam 2 mg, Loprazolam 1 mg, Lorazepam 2.5 mg, Zolpidem 10 mg
TIME ELAPSED SINCE PRESCRIPTION OF THE BZRA IN PROGRESS	[*n* (%)]
[0–5] years	3 (20.0%)
[6–10] years	10 (66.7%)
>10 years	2 (13.3%)
**Descriptive Statistics:** Range [1.22 years]; Median 8 years; Mean 7.6 years	
PREVIOUS INTAKE OF OTHER BZRA (*n* (%))	9 (60.0%)
REASON FOR TAKING BZRA (according to the participant)	[*n* (%)]
Related to insomnia	7 (46.7%)
Related to anxiety	7 (46.7%)
Related to insomnia and anxiety	1 (6.7%)
FREQUENCY OF MEDICATION INTAKE	[*n* (%)]
Daily	10 (66.7%)
On-demand	5 (33.3%)
PREVIOUS DISCONTINUATION ATTEMPTS [*n* (%)]	6 (40.0%)

## Data Availability

The data presented in this study are available on request from the corresponding author due to privacy and ethical reasons.

## Abbreviations

The following abbreviations are used in this manuscript:

BZRA	Benzodiazepine agonist receptors
COREQ	Consolidated Criteria for Reporting Qualitative Studies
DGS	Directorate-General of Health (Portugal)
ICPC-2	International Classification of Primary Health Care
MIM@UF	Module for Information and Monitoring of Functional Units

## Appendix A

**Table A1:** Themes and subthemes identified as barriers to discontinuation of BZRA (part 1/3).

**1. Patient characteristics**
**a. Prejudice of age**
“I think so (…) at this age, what should you do? On the couch… One no longer gets tired, doesn’t do anything anymore, at night one can’t sleep (…)” [M2]
“Yes, I think so [*age interferes with the possibility of stopping BZRA]* (…) For me it will no longer help, but it might help my children, who are in their 50 s…” [W2]
“I think that with our age, no one else [*could help stop BZRA*]” [W3]
“When we’re young, we want to sleep. Now at old age we don’t, we think a lot, we no longer sleep (…)” [M4]
**2. Clinical factors**
**a. Chronicity of the psychological condition related to the use of BZRA**
“Maybe no one [*could help stop BZRA*] because I’m a very nervous person … And for many years now.” [W1]
“These things are always inside me, all of this is always inside me… I don’t believe it will ever come out.” [W4]
“I’ve told this several times to my doctor and she says what I have it’s psychological. So, be it… I can’t make it!” [W6]
“I also wonder, why can’t I sleep? I wonder so, I don’t know! (…) And yet it’s been like this for such a long time…” [W9]
**b. Bad sleep habits**
“I never go to bed before midnight, that’s that! It’s the habitude we get from a lifetime and I’m not too keen on going to bed early.” [W4]
“And then if I come down here at 10 or 11 o’clock, I sit there a little bit on the couch, that’s when I get to sleep. (…) If I go to bed, I don’t sleep… But here on the couch, I’m watching TV, I want to watch the news, and then I let myself asleep!” [M4]
**c. Perception of brain/nervous system vulnerability**
“(…) I think this pill is just for the nervous system, because one is changed in… our nervous system… It happens that of all a sudden one starts thinking about the things you shouldn’t think about…” [M1]
“(…) we have already a certain age, therefore we have a weakest brain, I am aware of that. And then the brain will get those things, always… Even if we want to forget, we can’t.” [W3]
“This is already the nervous system I have.” [M4]
*Question:* “… we are talking about such difficulties…” *Answer:* “… that will affect my brain…” *Question:* “… that you fear would come to light if you didn’t have this medicine…” *Answer:* “Yes, I’m afraid… I’m sure of that.” [W5]

**Table A2:** Themes and subthemes identified as barriers to discontinuation of BZRA (part 2/3).

**3. Medication-related factors**
**a. Habituation and concern with withdrawal symptoms**
“Possibly, if one keeps not resting as he should, not sleeping as he should, headaches may arise, many problems may arise.” [M2]
“(…) if I don’t take it… I suddenly feel the difference (…) I don’t sleep (…) my head goes around a lot (…) I get dizzy, I feel bad.” [W1]
“When I was younger, I didn’t really want to get used to it, but now that’s how I feel good” [W2]
“If I don’t take it, I’ll spend a whole night… Because once there weren’t these pills, (…) they prescribed me some others (…), I spent a whole week without sleeping!… I even thought I went mad, I couldn’t sleep either night or day” [W5]
“I feel so bad, so bad (…) It’s like I miss something!” [W6]
“I think this drug should also give habituation, certainly, like any other alike it, doesn’t it?… The thing is I feel good having it and I don’t think it will do to me anything opposite of what I intend from taking it (…) I’m so used taking that pill and feeling good with it, I really don’t know what would happen if I had to stop it.” [W7]
“I think I would feel really bad… I wouldn’t sleep, I’d argue more, I’d get angrier (…) I’d feel out of control.” [W10]
**b. Valuing the therapeutic benefit of BZRA**
“(…) one takes the sleeping pill to rest a little bit, to keep that thought away from your mind.” [M1]
“(…) at night you can’t sleep if you don’t take the proper medicine.” [M2]
“It’s to rest my brain so that next morning I’m able to carry my life forward. (…) Because when I take this precious pill, after half an hour I’m asleep… If I don’t take it, I spend a whole night awake…” [W5]
“Because I’m doing really fine with it (…) I couldn’t sleep anymore, I couldn’t eat, I couldn’t do anything… I’ve been so bad, so bad…” [W6]
“(…) in a way, it calms me down to face situations that come across me (…) With this medicine, I can face things more lightly, in a better way, doing my normal life.” [W7]
“… I take it, that day… for that moment (…) And that’s it, after a while it does the effect I intended, and I carry on…” [W11]
**c. Disbelief in a non-pharmacological alternative**
“Without taking medication? No, no, no!… I don’t think it’s possible, no!” [W6]
“(…) that’s why I say it wouldn’t be worth another health professional in any other subject, either psychologist or whatever, because it really [*would always need a pharmacological alternative*]” [W7]
*Question:* “… Do you consider the possibility of an alternative which doesn’t involve medication?” *Answer:* “There are so many, aren’t there?… I could also train my mind, so I won’t have to deal with this, but no… Other than medicines, I don’t see any other way.” [W11]

**Table A3:** Themes and subthemes identified as barriers to discontinuation of BZRA (part 3/3).

**4. Context and external factors**
**a. Family and social context**
*Question:* “Therefore, having to deal with such a difficult situation for such a long time [*family members with alcohol problems*], you think that hardly anyone would be able to help you, is that right?” *Answer:* “I think so” [W1]
“Then I get anxious, and everything comes to my mind… The past, being alone, living with a retirement pension of less than five hundred euros…” [W5]
“… I’ve had a lot of problems in my family with people who have died, including my husband… and so it wasn’t easy … within four years I lost three people… Since then, there’s always this feeling remaining that you cannot carry on alone … having to deal with so many events, you see?” [W7]
“To reduce these medications … only if what I have in my place could improve enough, for me to get rid of… this grief I feel inside me.”
“…the health of those close to me concerns me a lot. (…) Because it’s a lot of accumulated stuff, and it turns out to be that the escape [*BZRA intake*] that makes me calm down a little bit. (…) This one of those situations which, even if we want to, we can’t make it otherwise… That’s a fact.” [W11]
**b. Community context/Prejudices towards mental health**
“Nobody took me to the psychiatrist, it was me who felt like I was going crazy. Since then, I start going there on my own, even now it is still so, no one goes with me, and no one forces me to go there. Because my ancestors, from my mother’s side, everyone died crazy. (…) And I’m very afraid of that.” [W5]
“… I think we all live very closed up… very closed up. And on mental health, or mental well-being, in my point of view, those things we all keep very much to ourselves. (…) If you don’t feel at ease, you keep it to yourself… Otherwise others may think you are crazy! So, it’s better not to say anything…” [W11]
**c. Lack of medical support**
*Question:* “Do you fear not having the necessary medical support for those eventualities?” *Answer:* “Yes, maybe. (…) I’m not sure of that.” [M3]
*Question:* “At that point would you be afraid of not having the necessary medical help?” *Answer:* “Yes, yes… Then I can be afraid of it.” [W6]
**d. Other anxiety inducers**
“Pain also interferes with these things… If you don’t have pain, you sleep better…But with pain… it’s a problem… Then it’s a headache, then it’s also those things in my head, vertigo… (…) It’s already the nervous system that I have.” [M4]
“Not to take it makes me feel bad and having to bother the others… (…) That’s the situation.” [W6]
“Because my heart beats very fast, I get tired afterwards… I feel so tired, I get very anxious… my hands freeze a lot, I get very anxious (…) Then I take that pill, that day, for that moment.” [W11]

**Table A4:** Themes and subthemes identified as facilitators for the discontinuation of BZRA (part 1/2).

**1. Motivation to stop BZRA**
“… to see if I could get rid of it. (…) I look forward to it … as long as I felt supported, informed, and so on…” [W5]
“One of two things should happen: either I had to mentalize myself I couldn’t take it, or I get another drug to replace it. (…) I think the first person which may help me must be myself. I’m the one who has to help myself.” [W11]
**2. Patient’s knowledge about BZRA**
**a. Negative/side effects of BZRA**
*Question:* “Who do you think could help you stop this medication, first of all?” *Answer:* “First of all… for me, I should go to a doctor … to realize if this medication could bring me trouble or not…” [M1]
**b. Benefits of discontinuing medication**
“One time I said to myself: “I don’t take them anymore! » It was even worse! I didn’t dream so many stuffs, but I couldn’t get myself asleep… But I didn’t dream so much, with this medicine you imagine things while you sleep…” [M4]
“I’m really sick with taking it… It’s not because it harms me, but still, I don’t know… This addiction, if I just could do without it, it would be good…” [W9]
“As it has its effect on me, it whets my appetite and that’s when I get to eat… And as I tend to put on weight, I think it even does me so, I can’t lose weight because of this medication.” [W10]
**3. Perception of BZRA insufficiency regarding to the therapeutic goal**
“Look, it has this effect: if I go to bed and don’t think about the pill, sometimes I even sleep well… It’s all psychological!… And if I go to bed and realize, “Hey! I didn’t take the pill! “, I can’t sleep!… I just have to take it!” [M4]
“This kind of medication also has a lot to do with our ability to fit things in our head… Because sometimes I think that when we take the pill, of course it does what it has to do in ourselves, but the fact that we know we are taking it also comforts us somehow…” [W11]
**4. Access to written information**
“The fact that we read something about the subject and think about it during that time… It’s already some help, I guess…” [M1]
“If it was something that I realize it would help me, and made me feel good, yes… It’s a means… It could be…” [M3]
“It’s quite useful… About this and about everything… Whether it is on paper, digital, or whatever, whatever informs you is welcome.” [W11]

**Table A5:** Themes and subthemes identified as facilitators for the discontinuation of BZRA (part 2/2).

**5. Access to alternatives**
**a. Pharmacological alternatives**
“(…) a medication that should be similar to that or that could replace that one.” [M2]
“There’s already so much you can take, natural products, from what I’m told… But I’ve never got into that yet… But maybe there are some other things at the pharmacy … A kind of supplement, something that could replace that pill but that was a natural product…” [W9]
**b. Non-pharmacological alternatives**
“If I stand a while without taking it, or if there was something else that the doctor should advise me: “For your health or for your age, I think the proper medicine is this…”, then I might even try other things instead.” *Question:* “What kind of “other things” could help other than medication?” *Answer:* “A psychologist. It could be a psychologist.” [W3]
“(…) a psychologist would have the knowledge to advise me on such a thing, I think so.” [W4]
**6. Time for decision-making**
*Question:* “Would this be a situation in which you think that being given some time to consider this possibility could be important?” *Answer:* “Yes, for me it would be.” [M1]
“Yes, only if I was given the time to think even a little bit… Other than that, I couldn’t, no, I couldn’t…” [W6]
“Gradually, little by little… I think so, it would be better.” [W9]
**7. Attitudes of health professionals**
**a. Competencies**
“(…) because I’m not going to address to any person without having the certainty and conviction of what I’m going to do and the answer I’m going to get.” [M2]
“Yes, a psychologist would have the knowledge to advise me on such a thing, I think so.” [W4]
“Any health professional could be helpful… Because they are aware of this matter, aren’t they?” [M4]
*Question:* (…) so, considering this possibility, you think only a specialized doctor could help?” *Answer:* “With all due respect, I think a general practitioner wouldn’t be able to help on this.” [W6]
“My family doctor would be the right person to advise me on this, of course.” [W8]
**b. Availability**
“If in one of my regular medical appointments, (…) my doctor says to me “you should stop taking this medication, because this may be harming you for this and for that reasons”, then automatically I shall stop taking it…” [M1]
